# The Importance of Gene Duplication and Domain Repeat Expansion for the Function and Evolution of Fertilization Proteins

**DOI:** 10.3389/fcell.2022.827454

**Published:** 2022-01-27

**Authors:** Alberto M. Rivera, Willie J. Swanson

**Affiliations:** Department of Genome Sciences, University of Washington, Seattle, WA, United States

**Keywords:** gene duplication, fertilization, subfunctionalization, neofunctionalization, sperm, egg, reproduction

## Abstract

The process of gene duplication followed by gene loss or evolution of new functions has been studied extensively, yet the role gene duplication plays in the function and evolution of fertilization proteins is underappreciated. Gene duplication is observed in many fertilization protein families including Izumo, DCST, ZP, and the TFP superfamily. Molecules mediating fertilization are part of larger gene families expressed in a variety of tissues, but gene duplication followed by structural modifications has often facilitated their cooption into a fertilization function. Repeat expansions of functional domains within a gene also provide opportunities for the evolution of novel fertilization protein. ZP proteins with domain repeat expansions are linked to species-specificity in fertilization and TFP proteins that experienced domain duplications were coopted into a novel sperm function. This review outlines the importance of gene duplications and repeat domain expansions in the evolution of fertilization proteins.

## Introduction

The fertilization of oocytes by sperm is an essential function in sexual reproduction, and multiple stages of the fertilization cascade have been described (Vacquier, 1998). First the sperm is drawn to the egg through chemotaxis (Ramírez-Gómez et al., 2019), and it then binds to the egg and releases proteins stored in the acrosome. The sperm then passes through the glycoproteinaceous egg coat (Monne et al., 2008; Wilburn and Swanson, 2016) (named Zona Pellucida in mammals), and proceeds to the oocyte cell membrane to initiate fusion (Siu et al., 2021). Understanding fertilization requires knowledge of both these broad steps of the fertilization cascade and the molecular mechanism underlying them. Research into the evolution and function of gametic proteins has implications for the development of novel contraception or treatments for unexplained human infertility (Gelbaya et al., 2014).

Many fertilization proteins are members of gene families that result from whole gene duplication events, which is a common mechanism for gene birth (Hughes, 1994). There has been extensive research into the relationship between gene duplication and other aspects of reproductive biology, including the neuroendocrine control of reproduction (Dufour et al., 2020), protease activity in the female reproductive tract (Kelleher et al., 2007; Kelleher and Markow, 2009), the resolution of sexual conflict (Gallach et al., 2010, 2011; Connallon and Clark, 2011; Gallach and Betrán, 2011), and hybridization barriers (Ting et al., 2004). This review specifically focuses on our growing knowledge of duplicated protein families implicated in fertilization. These proteins include the Izumo1 and Juno pair of interacting proteins, which each arose from independent gene duplication events and are essential to gamete membrane fusion function in mammals (Bianchi et al., 2014). DCST1 and DCST2 are paralogous proteins expressed in the sperm membrane of some bilateral animals, that are essential for fertilization (Inoue et al., 2021a, 1). Other duplicated proteins that act in fertilization include ADAMs (Primakoff and Myles, 2000; Civetta, 2003; Finn and Civetta, 2010), CRISPs (Busso et al., 2007; Da Ros et al., 2008; Gibbs et al., 2011; Maldera et al., 2014), Catspers (Clapham and Garbers, 2005; Navarro et al., 2008; Speer et al., 2021), and PKDREJ on the male side (Sutton et al., 2008), and tetraspanins (CD9,CD81) (Le Naour et al., 2000, 9; Miyado et al., 2000; Frolikova et al., 2018) and EBR1 on the female side (Kamei and Glabe, 2003; Hart, 2013). Genomic resources suggests that most of these families (ADAMs, tetraspanins, EBR, PKRDEJ, Catsper) have orthologs in other bilateral animals, while CRISP has orthologs in animals and in yeast (Howe et al., 2021).

**FIGURE 1:**
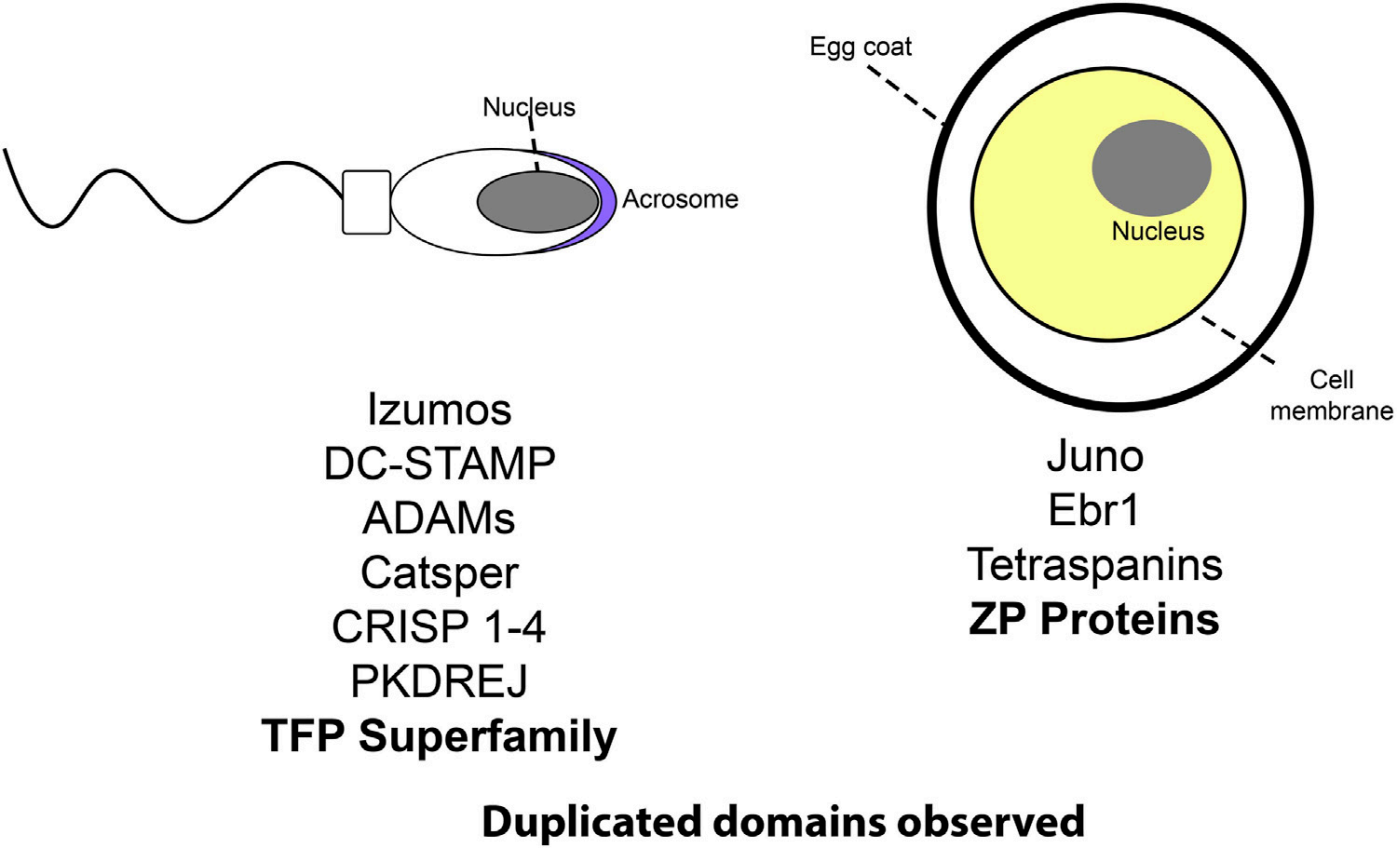
A cartoon schematic listing several protein families involved in reproduction. Those with notable repeat expansions are bolded.

Duplicated genes can experience further structural diversification, such as the duplication of individual functional protein domains. Proteins containing tandemly duplicated domains constitute a small, but significant portion of the genome (Han et al., 2007; Nacher et al., 2010). Independent tandem duplications of individual functional domains is also a recurrent trend in some protein families (TFP,ZP) (Galindo et al., 2002; Aagaard et al., 2010; Doty et al., 2016). There are several families of reproductive proteins on both the sperm and egg that show a history of being coopted from non-reproductive functions (Figure 1). Three finger proteins (TFPs) have been frequently coopted for fertilization including SPACA4 in tetrapods, Bouncer in fish, and multiple classes of sperm proteins in plethodontid salamanders (PMF, SPFs) (Doty et al., 2016; Fujihara et al., 2021). Salamander SPFs have a duplicated three finger protein domain, and have evolved structural modifications to those domains (Doty et al., 2016). Similarly, the family of ZP proteins (named after the Zona Pellucida), essential components of egg coats across vertebrates and invertebrates (Wilburn and Swanson, 2016), show evidence of independent expansions of ZP-N domains in different lineages (Liang and Dean, 1993; Galindo et al., 2002). These highlight the role of gene duplication and repeat domain expansions in fertilization. An observed trend is rapid sequence evolution in reproductive proteins (Swanson and Vacquier, 2002), and newly duplicated domains can provide novel substrates for evolving new functions at multiple stages of the fertilization cascade.

The role of duplications in genome evolution is well documented across the tree of life. (Kondrashov et al., 2002; Conant and Wolfe, 2008). Gene duplication (Ponting, 2008) is an important source for new genetic material that facilitates biological innovation. The duplication and differentiation of genomic regions has been linked to the evolution of modularity in organisms (Wagner et al., 2007). Modularity is an abstract concept in which part of an organism (such as a network of protein interactions) functions largely autonomously relative to other aspects of the organisms’ biology (Wagner and Altenberg, 1996; West-Eberhard, 2005). Duplicated genes can participate in existing modular protein interaction networks, which facilitates increasing biological complexity of these networks (Wagner et al., 2007). Such increases in modular network complexity through gene duplication has been linked to adaptations in humans (Perry et al., 2007). Duplicated functional domains can similarly contribute to the evolution of biological complexity. This review will discuss both whole gene duplications and within gene domain duplications, and their role in the evolution of reproductive functions.

**FIGURE 2:**
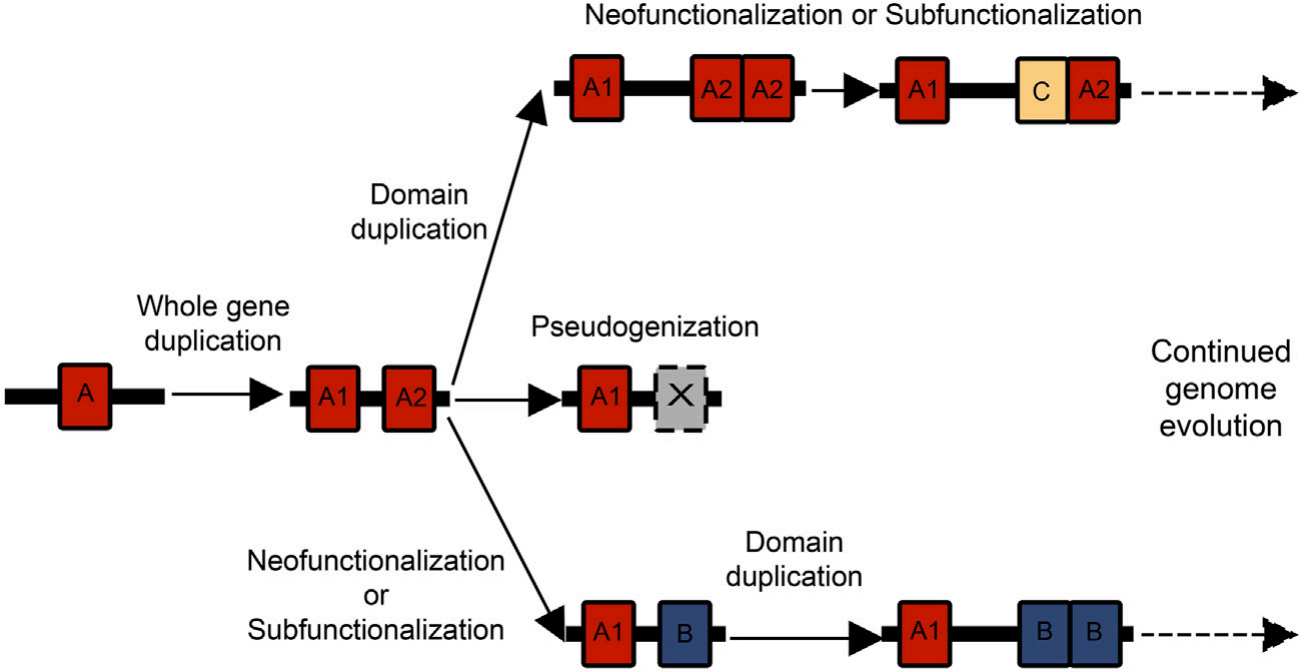
There are multiple possible combinations of whole gene and domain duplications that can birth new genes and functional domains. Often a whole gene duplication begins the process, and then one of the gene duplicates experiences a domain expansion. These genes can then act as substrates for further duplication and neofunctionalization or subfunctionalization events.

When genes duplicate they experience one of three possible fates: pseudogenization, subfunctionalization, and neofunctionalization (Walsh, 2003; Innan, 2009). Due to redundancies in function, the duplicated gene may no longer experience conservation and accumulate silencing mutations, resulting in a non-coding “pseudogene” (Figure 2). New mutations are frequently deleterious, so pseudogenization is hypothesized to be the most common fate of duplicated genes (Lynch and Conery, 2000). However, the other two fates of duplicated genes (subfunctionalization and neofunctionalization) are common mechanisms for biological innovation. Under neofunctionalization, one gene copy maintains its original function while the other experiences positive selection and evolves a novel function. While under subfunctionalization, both copies parse the original function, and neither gene is sufficient (Walsh, 2003; Innan, 2009).

Tandem duplications of individual protein domains within a gene can add greater complexity to the duplication process. Paralogous genes experiencing relaxed selection can have greater freedom for tandem domain duplications. There is strong research interest in the mechanisms underlying domain repeat expansions and how they affect the evolution of protein families (Björklund et al., 2005, 2006; Vogel et al., 2005; Weiner et al., 2006; Moore et al., 2008; Buljan and Bateman, 2009). Repeats can experience concerted evolution where they maintain a high degree of sequence identity (Elder and Turner, 1995; Liao, 1999), through unequal recombination and gene conversion (Schimenti, 1999). Under this scenario, the repeat expansion of highly identical domains is itself an innovation that could allow proteins to evolve novel functions. A repeat domain expansion could also affect dosage or protein interaction networks. Repeated domains could similarly differentiate in amino acid sequence, leading to neofunctionalization or subfunctionalization with the original domain. There are many possible orders and combinations of whole gene duplications and domain duplications that can contribute to the expansion of gene families (Figure 2). The process by which duplicate genes are maintained and experience subfunctionalization or neofunctionalization has been characterized under the duplication-degeneration-complementation model (DDC) (Force et al., 1999). While most classical population genetics models (Walsh, 2003; Innan, 2009) primarily discuss the effect of silencing or beneficial mutations on coding regions, the DDC model focuses on the effect of mutations on regulatory regions and subfunctionalization. Essentially, mutations that can silence certain regulatory regions in a duplicate gene can lead to the two genes partitioning expression and eventually function (Force et al., 1999). Other models have suggested subfunctionalization is primarily important as a transition phase to neofunctionalization (Rastogi and Liberles, 2005). The mechanisms of subfunctionalization and neofunctionalization remain a subject of rich debate, and concepts like the DDC model could have ramifications for protein evolution.

Subfunctionalization and neofunctionalization are foundational to the evolution of increased complexity in genomes and protein networks, and it is worth examining their particular importance in fertilization. Fertilization proteins are some of the most rapidly evolving proteins in genomes, as evidenced by high amino divergence (Swanson and Vacquier, 2002). Their rapid evolution is likely driven by factors such as sexual conflict and molecular arms race dynamics between gametes, which can also contribute to the maintenance of fertilization barriers between species (Gavrilets and Waxman, 2002; Gavrilets, 2014). The general trend of rapid evolution in reproductive proteins could facilitate the subfunctionalization or neofunctionalization of domains.

## Izumo/Juno

The fusion of sperm and egg is necessary for fertilization, but there are only a few known pairs of interacting gametic proteins identified at this stage (Wilburn and Swanson, 2016). After years of research the interacting pair Izumo1 and Juno were identified in mammals (Bianchi et al., 2014). Izumo1 is the sperm expressed protein that mediates fusion (Inoue et al., 2005), and it interacts with the egg surface bound folate receptor 4 (known as Juno) (Bianchi and Wright, 2014). Izumo1 and Juno are each part of protein families with multiple paralogues, but only the Izumo1/Juno pair is capable of interacting (Bianchi et al., 2014). There are four members of both the Izumo (Ellerman et al., 2009) and folate receptor families (FOLR) in mammals (Elwood, 1989; Shen et al., 1994; Spiegelstein et al., 2000; Petronella and Drouin, 2014). Despite being part of the folate receptor family, Juno does not actually bind folate, exemplifying how a single member of this gene family has been coopted for a novel reproductive function (Bianchi et al., 2014).

While Juno represents a clear cooption into fertilization, the evolution of the Izumo gene family could also present an interesting example of neofunctionalization. Izumo1-4 all have a highly structurally conserved Izumo domain, but Izumo1 and Izumo4 have a shared pair of β-strands extending from this domain. Izumo1 experienced further structural modifications, as its β-strand extensions act as a hinge between the Izumo domain and a coopted immunoglobulin-like domain (Aydin et al., 2016; Ohto et al., 2016). Such substantial structural changes could be important for the protein’s ability to bind Juno. Research into other Izumo proteins suggests their involvement in fertilization. Izumo1-3 are transmembrane testis expressed proteins (Ellerman et al., 2009), while Izumo4 lacks a transmembrane domain and is expressed in the acrosome (Guasti et al., 2020). Izumo3 shows evidence of positive selection (Grayson and Civetta, 2012), and is necessary for sperm acrosome formation (Inoue et al., 2021b). The parallel histories of structural modifications in Izumo1 and Juno allowed for this essential interaction to evolve.

**FIGURE 3:**
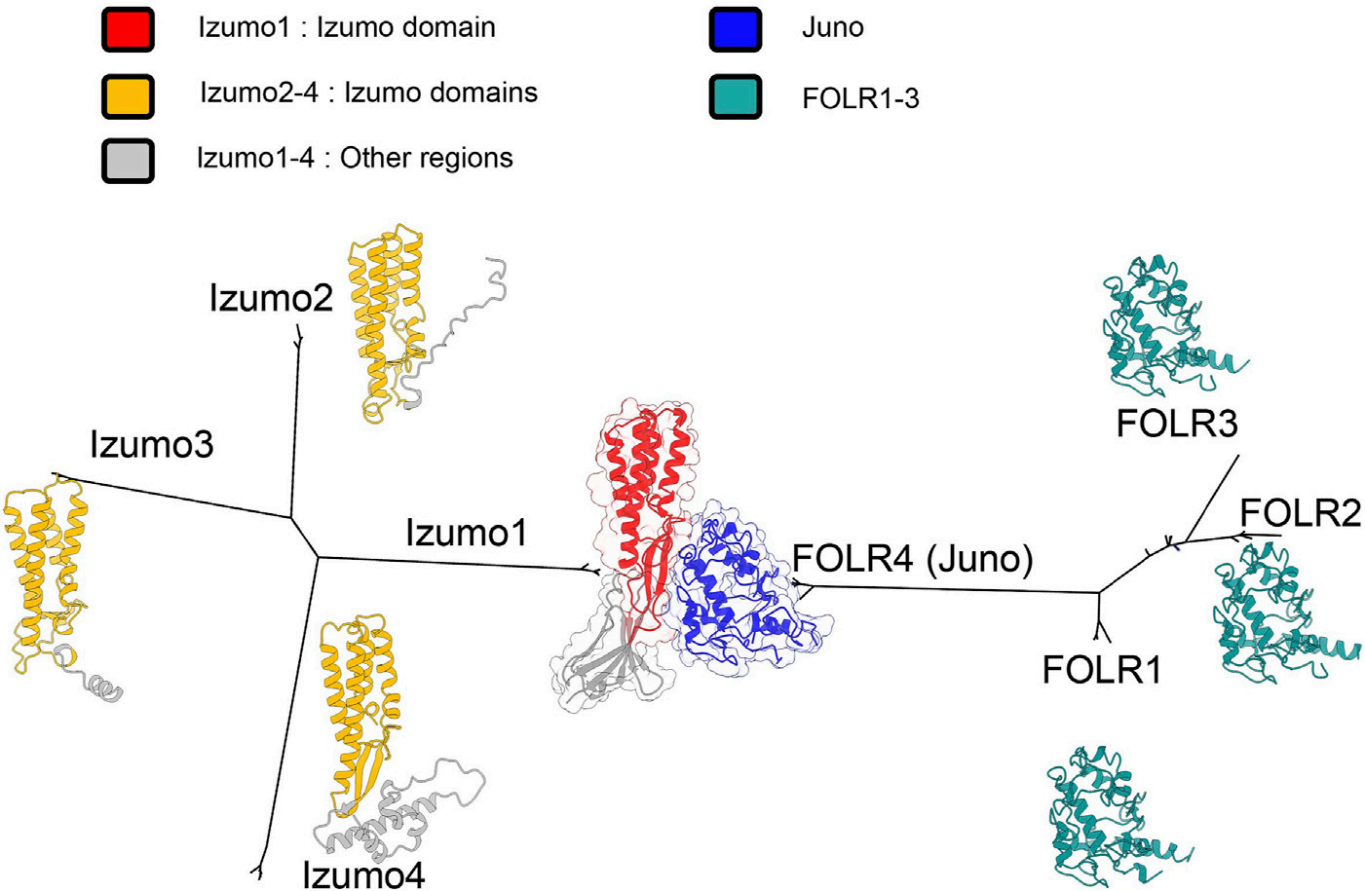
Unrooted maximum likelihood phylogenies for *Izumo* and *FOLR* gene families in a subset of primates, based on multiple sequence alignments (Katoh and Standley, 2013; Kozlov et al., 2019). Both gene families independently duplicated, but *FOLR4* was coopted to bind *Izumo1*. Crystal structures have been obtained for the Izumo1-Juno complex (Aydin et al., 2016). For other proteins, alphafold predicted structures were used (Jumper et al., 2021). Using predictions of signal peptides and transmembrane domains, and secondary structural alignments, we identified shared izumo domains (Sonnhammer et al., 1998; Krogh et al., 2001; Almagro Armenteros et al., 2019).

The relationship between Izumo1, Juno and their paralogs is highlighted by our phylogeny (Figure 3), which contains a long branch leading to Juno (FOLR4). This could reflect the rapid accumulation of mutations in the Juno branch as it was coopted to bind Izumo1 during gametic membrane fusion. Crystal structures confirm that 1:1 binding complexes form between Izumo1 and Juno (Aydin et al., 2016; Ohto et al., 2016). The adhesion of Izumo1 and Juno is conserved in mammals, and after the adhesion event Juno is released from the egg’s surface in vesicles and may act to bind and neutralize acrosome reacted sperm (Bianchi et al., 2014). In mammals, this interaction functions as a block against polyspermy (Bianchi and Wright, 2014). Blocks to polyspermy are essential, because eggs that fuse with multiple sperm are not viable and mammalian blocks to polyspermy exist at both the cell membrane (Evans, 2020) and egg coat (Fahrenkamp et al., 2020).

Mutations to residues conserved in mammals greatly reduce binding, highlighting that particular changes to amino acid sequence and protein structure facilitated the neofunctionalization of Juno (Aydin et al., 2016). The more variable structural features (Ohto et al., 2016) in Juno may be important for the species-specificity of its binding to Izumo1 (Bianchi et al., 2014; Bianchi and Wright, 2015; Han et al., 2016). Comparative genetic analyses identify positive selection in a subset of mammals (Laurasiatheria) (Grayson and Civetta, 2012), and that Juno is likely rapidly coevolving with Izumo1, which contributes to the specificity of their interactions (Grayson, 2015). This specific binding is essential to both Juno’s function in initiating membrane fusion, and the post-fusion neutralization of acrosome-reacted sperm (Wright and Bianchi, 2016).

## DCST

While Izumo1 and Juno are thought to initiate the complex molecular process of gametic membrane fusion in mammals, recent transgenic experiments and complementation studies have demonstrated that DCST1 and DCST2 are also essential (Inoue et al., 2021a). The DCST1/2 proteins are expressed on the sperm surface, and contain variable (4–6) transmembrane helical domains (DC-STAMP) (Inoue et al., 2021a, 1). DC-STAMP (dendritic cell specific transmembrane protein) refers to both the name of the domain and one of the proteins that contains this domain (Hartgers et al., 2000). The originally identified DC-STAMP protein has four transmembrane domains (Hartgers et al., 2001), and it is highly expressed in myeloid dendrocytes (Hartgers et al., 2000, 2001; Eleveld-Trancikova et al., 2005, 2008). The expression of DC-STAMP has been induced in macrophages (Staege et al., 2001) and osteoclasts (Nomiyama et al., 2005). This broad array of functions has motivated much research into the molecular mechanisms of DC-STAMP interactions, which has supported a role in osteoclast fusion (Kukita et al., 2004; Yagi et al., 2005; Jansen et al., 2009). There is also evidence of DC-STAMP related signaling in immune response (Nair et al., 2016). Along with these other diverse functions, it seem that DC-STAMP domains have been coopted into an essential role in sperm-egg membrane fusion.

**FIGURE 4:**
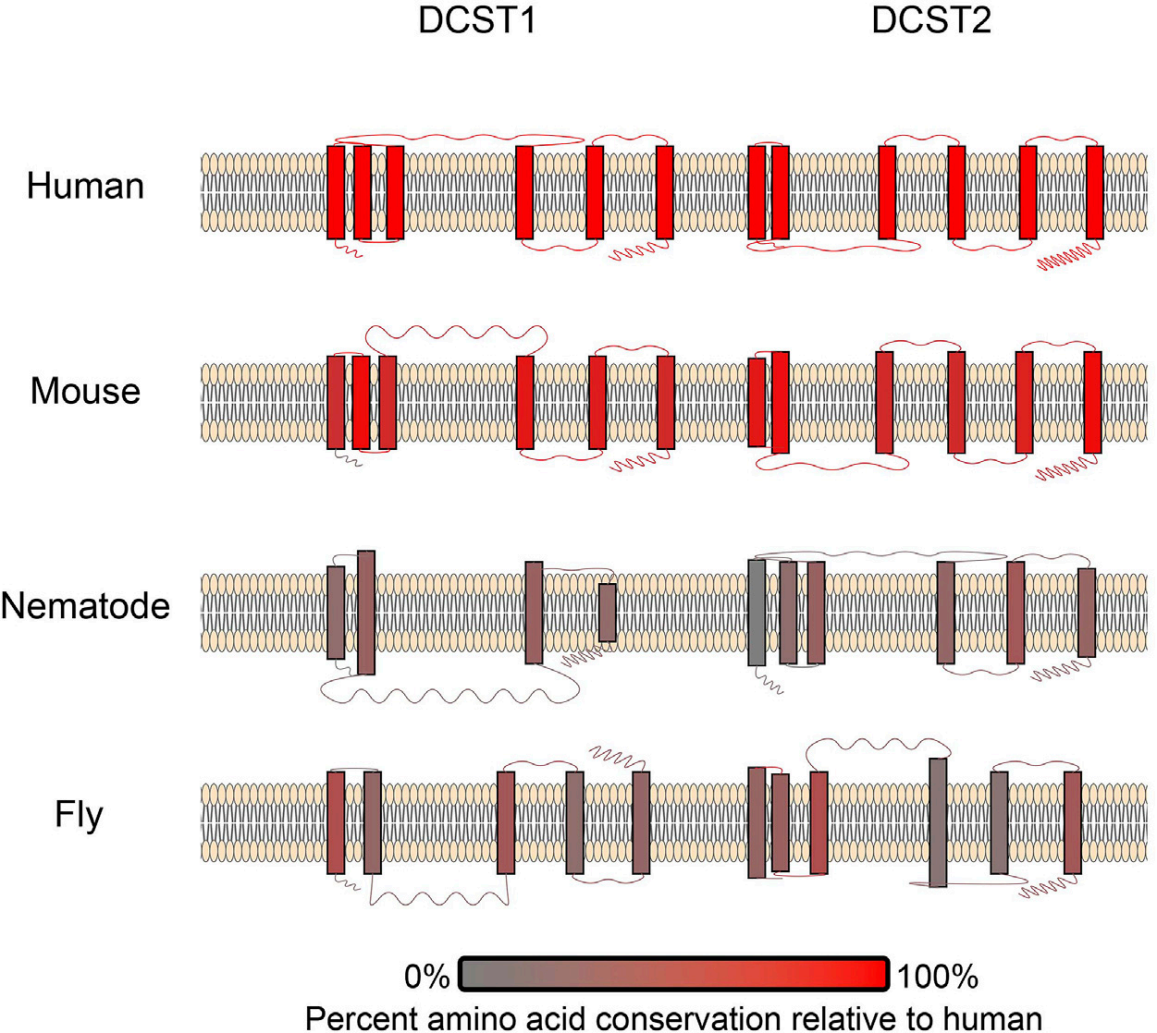
A schematic of DCST1/2 proteins in multiple species. The number of transmembrane domains and loop lengths differ across species. Transmembrane domains and loops are colored based on conservation (Pei et al., 2008), where red coloration signifies amino acid conservation relative to humans. Therefore, the human examples are all red.

DCST1/2 are the first known essential fertilization factors that are conserved in both vertebrates and invertebrates (Inoue et al., 2021a). DCST1/2 orthologues have been identified in both *Caenorhabditis* and *Drosophila* (Kroft et al., 2005; Wilson et al., 2006, 2018), which is the first known example sperm related factors being conserved this broadly across vertebrates and invertebrates (Inoue et al., 2021a, 1). However, there has been extensive structural diversification of these DCST1/2 across animals (Figure 4), especially between invertebrates and vertebrates. The low sequence identity of DCST1/2 proteins across animals, makes the conservation of reproductive function all the more remarkable. The ubiquitin ligase activity of DCST1 (Nair et al., 2016) raises questions about the function of DCST1/2 in sperm. There is intense research interest into the signal activity of long non-coding RNA produced by DCST1 and its effect on cancer cell progression (Hu et al., 2020; Ai et al., 2021, 1; Wang et al., 2021). More investigation is necessary to understand the function of DC-STAMP domains in a broad range of signaling networks, and how they were neofunctionalized in sperm DCST1/2.

## ZP Domains

ZP proteins are an essential class of egg coat proteins. An important feature of ZP proteins is the ZP module that consists of two domains, ZP-N and ZP-C, named after their relative N-terminal and C-terminal positioning. ZP-N and ZP-C domain are immunoglobular domains with characteristic patterns of disulfide bonding and β-sheets (Bokhove and Jovine, 2018), and likely resulted from an ancestral domain duplication. The variability in amino acid sequence, disulfide placement, and loop structures between ZP-N and ZP-C (Lin et al., 2011) suggests differences in their biological function and evolutionary history.

ZP-N domains are of particular interest, because they form asymmetric dimers with their β-sandwich edges which are believed to promote polymerization between ZP modules (Jovine et al., 2002; Wilburn and Swanson, 2017; Bokhove and Jovine, 2018). There are several ZP proteins identified in vertebrates (ZP1-4, ZPAX and ZPD), and there appears to be a history of lineage specific gain and loss of ZP proteins among vertebrates (Galindo et al., 2002; Conner et al., 2005; Goudet et al., 2008; Claw and Swanson, 2012; Meslin et al., 2012; Shu et al., 2015; Killingbeck and Swanson, 2018). Like other families discussed in this review, there also multiple ZP proteins with non-reproductive functions (e.g., uromodulin and tectorin-alpha) (Legan et al., 1997; Brunati et al., 2015; Bokhove et al., 2016). This may be another example of domains being coopted into a reproductive function, and ZP-N polymerization domains may be important for egg coat assembly and structure.

**FIGURE 5:**
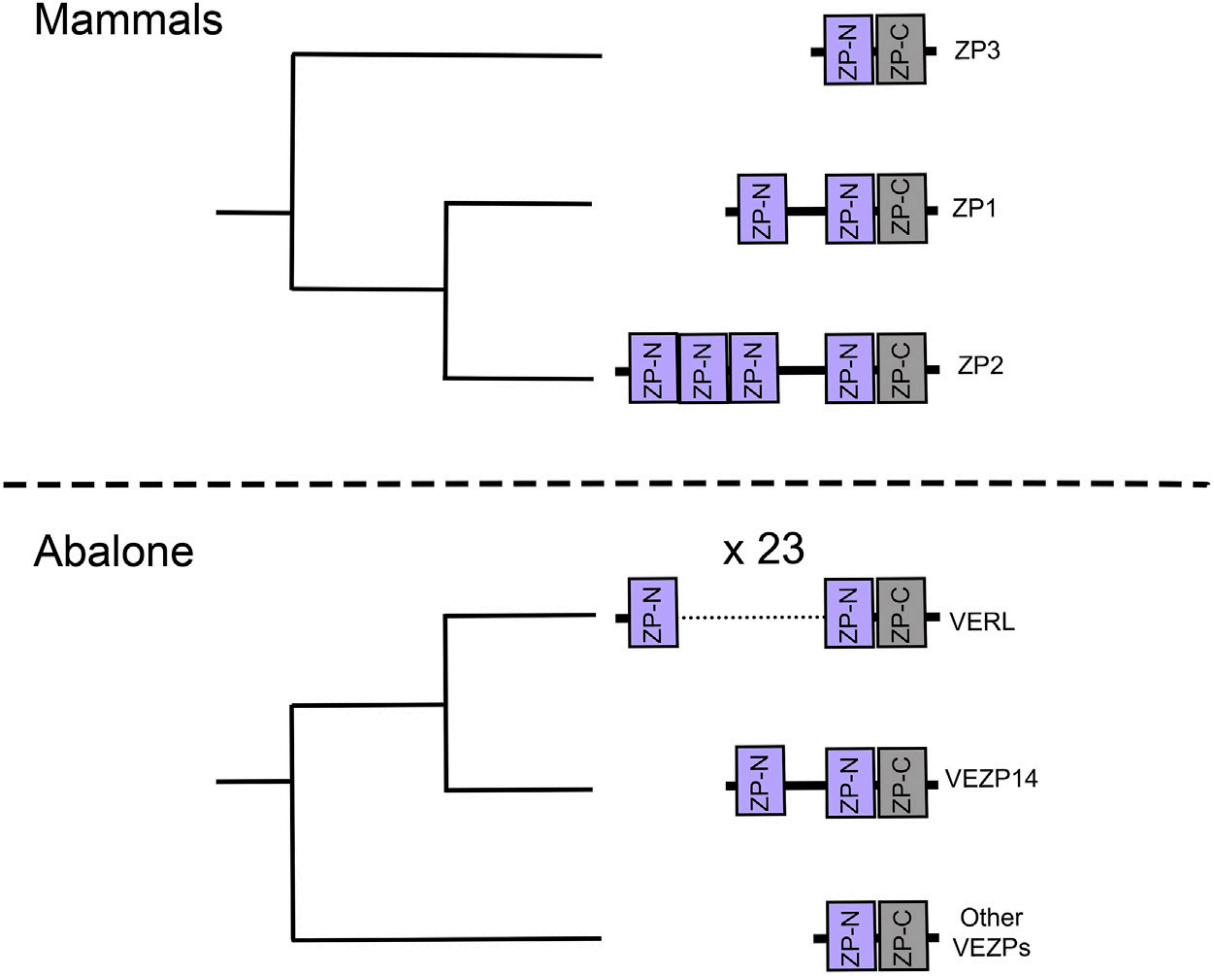
Cladograms of ZP-N proteins are based on phylogenies from the literature (Aagaard et al., 2010; Claw and Swanson, 2012). These suggest independent repeat expansion of the ZP-N domain in both abalone and human egg coat genes.

Not only has gene duplication produced an assortment of ZP proteins, there are also examples of independent repeat expansions of ZP-N in both vertebrates and invertebrate egg coat proteins (Figure 5). Some have only one additional ZP-N domain, but there are more dramatic repeat expansion like mammalian ZP2 (4 ZP-Ns) and abalone VERL (23 ZP-Ns) (Galindo et al., 2002). This process of domain duplications helped contribute to the diversity of ZP proteins. Given the ability of ZP-N domains to dimerize (Jovine et al., 2002; Bokhove and Jovine, 2018; Litscher and Wassarman, 2020), their duplications could create opportunities to evolve novel binding functions. Proteins with duplicated ZP-N domains, such as mammalian ZP2 and abalone VERL, are thought to be essential for species-specific in fertilization (Avella et al., 2013, 2014; Raj et al., 2017). Species-specificity in abalone is associated with the coevolution between VERL and the sperm protein lysin (Galindo et al., 2003; Clark et al., 2009), suggesting a cooption of ZP-Ns in sperm-egg interactions during egg coat dissolution.

Neofunctionalization of ZP-N domains can also drive new interactions between ZP proteins, such as the evolution of essential intermolecular crosslinks (Nishimura et al., 2019), which affect the physical assemblage of proteins in the supramolecular structure of the egg coat. Indeed, mouse research has suggested the importance of egg coat supramolecular structure in fertilization (Rankin et al., 2003; Avella et al., 2013). The structure of the egg coat is also important for the oocyte’s ability to block polyspermy. Protein cleavage of ZP2 is thought to initiate other egg coat structural modifications, which “harden” the egg coat and prevent sperm binding (Bleil et al., 1981; Gahlay et al., 2010; Fahrenkamp et al., 2020). Gene and domain duplications has produced a family of ZP proteins that contribute to the egg coat supramolecular structure, and are involved in both sperm recognition and polyspermy avoidance.

## TFP Superfamily

**FIGURE 6:**
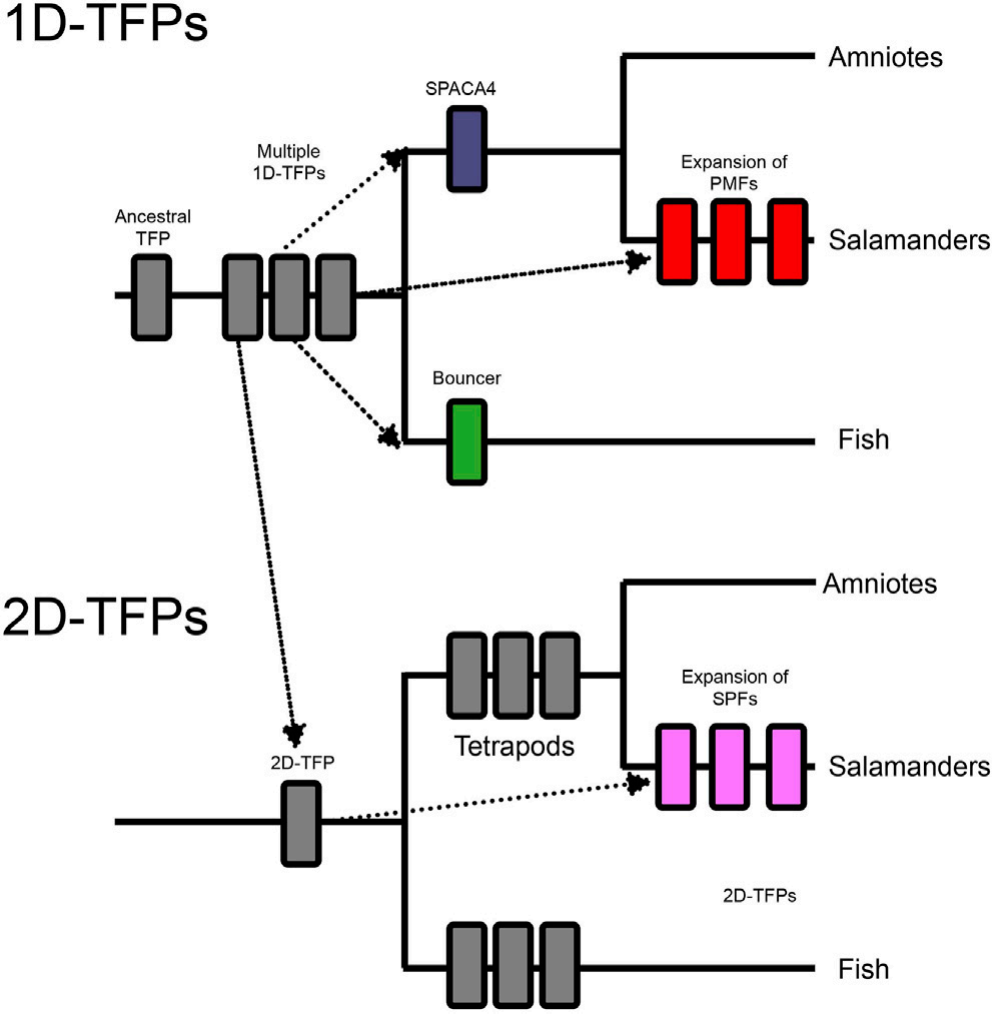
These two cladograms outline the whole gene and domain duplications within the three finger protein superfamilty (TFPs) and their expansions into reproductive systems. An ancestral single domain TFP (1D-TFP), duplicated into multiple vertebrate 1D-TFPs, and also had a domain level duplication which created a lineage of two TFP domain proteins (2D-TFPs). The 1D-TFPs produced tetrapod SPACA4, fish Bouncer, and multiple salamander PMFs. The 2D-TFPs also duplicated throughout vertebrates including salamander SPFs. Both salamander PMF and SPF protein families include both sperm and pheromone expressed members (Wilburn et al., 2022).

Three finger proteins are defined by their TFP domains, which have a characteristic disulfide bonding pattern and fold (Galat, 2008; Galat et al., 2008). The broader TFP protein superfamily also includes proteins with structurally modified TFP-like domains (Galat, 2015). While TFPs were originally identified in snake toxins (Low et al., 1976; Tsernoglou and Petsko, 1977), members of the TFP superfamily have been to coopted for reproductive functions into sperm (SPACA4, PMFs, and SPFs), egg (Bouncer), and pheromones (PMFs, and SPFs) (Doty et al., 2016; Fujihara et al., 2021; Wilburn et al., 2022) (Figure 6). Bouncer plays a role in species-specific sperm-egg fusion in teleost fish (Herberg et al., 2018), which raises questions about how other TFPs may function in fertilization. The TFP superfamily includes both soluble and membrane bound proteins, and has great functional diversity across many tissues and taxa (Alape-Girón et al., 1999; Tsetlin, 1999; Kini, 2002; Nirthanan et al., 2003; Kessler et al., 2017). Similar to ZP proteins, we observe a history of gene duplication, repeat expansion of domains, and functional diversification of TFP containing proteins.

An ancestral TFP protein experienced gene duplication to produce an assortment of single TFP-like domain proteins (1D-TFPs). One of these TFP genes experienced a tandem domain expansion to produce the ancestor of proteins with two TFP-like domains (2D-TFPs). Three independent cooption events have produced TFPs in gametes (Figure 6). A cooption of 1D-TFPs occurred in the ancestor of tetrapods and produced both Bouncer in fish, and SPACA4 in amniotes (Figure 6). Despite their protein homology, Bouncer is egg expressed while SPACA4 is sperm expressed and it is implicated in interactions between the sperm and egg coat (Fujihara et al., 2021), highlighting the functional diversification of TFPs. Another independent cooption of 1D-TFPs resulted in the sperm expressed plethodontid modulating factor (PMFs) salamanders, which extensively duplicated producing a diverse family of reproductive molecules (Wilburn et al., 2012, 2014, 2017; Doty et al., 2016). Salamander PMFs are hypervariable proteins expressed in multiple tissues, and while they are structurally similar to other TFPs, they differ in loop length and disulfide bridge patterning, and show evidence of persistent diversification and positive selection (Palmer et al., 2010; Wilburn et al., 2012, 2014).

Among 2D-TFPs there was independent cooption into the sodefrin precursor-like factors (SPFs) of salamander sperm. SPFs then experienced their own history of gene duplications and radiation (Palmer et al., 2007). Both PMFs and SPFs experienced disulfide bond reshuffling relative to the canonical 1D-TFP and 2D-TFP binding patterns, and these changes reflect the neofunctionalization of these molecules (Doty et al., 2016). These striking examples of independent gene duplications and neofunctionalization for reproductive functions raises questions as to whether there a more additional unknown cooptions of TFPs, and whether some protein domains are more susceptible to cooption in diverse biological contexts.

Both PMFs and SPFs are highly duplicated protein families, with some members being coopted into pheromone function and others for sperm expression (Doty et al., 2016; Wilburn et al., 2022). As the sperm paralogs of PMFs and SPFs have only recently been discovered, functional studies have not yet been conducted. Male salamanders produce large number of PMFS and SPFs within their mental glands which promote ritual courtship behavior in females (Doty et al., 2016). Duplications of secreted male-expressed sperm proteins could have provided an evolutionary substrate to evolve new pheromones (Wilburn et al., 2022). Structural changes in PMFs and SPFs, such as disulfide shuffling, may contribute to new functions in both sperm and pheromones. The TFP’s superfamily’s history of gene duplication, domain duplication, and neofunctionalization provides a unique model for the evolution of large gene families involved in fertilization.

## Discussion and Conclusion

Within this review we discussed examples of duplicated gene families with roles in fertilization. Gene duplication and neofunctionalization is an essential process for the evolution of greater genomic and functional complexity in organisms. Duplicated paralogous genes have been coopted into both sperm (Izumo1, DCST1/2) and egg (Juno) proteins involved in gamete membrane fusion (Bianchi et al., 2014; Inoue et al., 2021a, 1). Domain duplications within paralogs is also observed in the TFP superfamily and ZPs and has allowed both groups of genes to adopt novel functions at multiple stages of fertilization. As seen with TFPs, duplication events are often followed by notable protein structural changes (Doty et al., 2016) which may be tied to their cooption for novel fertilization functions. It is intriguing to consider hypotheses that account for these patterns of gene family expansion and diversification common in reproductive molecules.

Duplication events can facilitate the rapid evolution and neofunctionalization observed in many families of fertilization proteins. This rapid evolution can also be influenced by multiple factors such as sexual conflict, polyspermy avoidance, or genetic drift (Vacquier et al., 1997). The necessity of pathogen avoidance or blocks to polyspermy can drive oocytes to evolve reduced sperm binding ability. The sperm would then coevolutionarily “chase” the egg, which can contribute to the rapid sequence evolution of gametic proteins, and to the species-specificity of these protein interactions (Gavrilets and Waxman, 2002; Gavrilets, 2014). The rapid evolution of reproductive proteins is explored in terms of amino acid mutations, but the repeat expansion of domains could also be part of this trend. Proteins with repeated domains could experience drift resulting in ever-changing molecular target, that interacting proteins must coevolutionarily chase (Vacquier et al., 1997).

Duplications of reproductive proteins can also contribute to the phenomenon of functional redundancy, in which two duplicated genes have partially overlapping functions and can compensate for each other’s loss (Kafri et al., 2009). Functional redundancy has been observed in the CRISP family of reproductive proteins (Curci et al., 2020), and this property could emerge in other large protein families. While functional redundancy seems like it would be temporary as duplicated genes subfunctionalized or neofunctionalized, it can be a surprisingly evolutionarily stable property. Functional redundancy could confer fitness advantages by maintaining the robusticity of protein interaction networks in spite of stochasticity of expression between cells (Kafri et al., 2009). The rapid evolution of other reproductive proteins in these networks could place even greater value on robustness and stability of essential functions. Robusticity in these protein networks is believed to reduce the fitness cost of new mutations, which would increase the “evolvability” of these proteins and facilitate functional innovation (Kirschner and Gerhart, 2008). The concepts of functional redundancy and robusticity of function may also apply to domain repeat expansions like the ZP-N domains of VERL. The processes of gene duplication, repeat domain expansion, structural modification, and neofunctionalization have been fundamental to the evolution of reproductive molecules across life.
